# A contribution to the understanding of ocular and cerebrospinal fluid dynamics in astronauts during long-lasting spaceflight

**DOI:** 10.3325/cmj.2021.62.420

**Published:** 2021-08

**Authors:** Darko Orešković, Milan Radoš, Marijan Klarica

**Affiliations:** 1 Department of Molecular Biology, Ruđer Bošković Institute, Zagreb, Croatia doresk@irb.hr; 2 Department of Pharmacology and Croatian Institute for Brain Research, Zagreb University School of Medicine, Zagreb, Croatia

Zhang and Hargens (1) reviewed the knowledge on visual impairment intracranial pressure (VIIP) syndrome in astronauts, a condition that arises as a consequence of long-lasting spaceflight. However, the etiology and pathophysiology of VIIP syndrome remain unclear and only hypothetical. The main reason is the absence of data regarding astronauts' intracranial (ICP) and intraocular pressure (IOP) during long-lasting spaceflights. It is believed that a crucial role in the pathogenesis of VIIP syndrome is played by chronic ICP/IOP mismatch, which is a consequence of chronically elevated ICP and which results in translaminar pressure gradient. A direct transmission of elevated ICP from the intracranial to the intraocular compartment through the perioptic subarachnoid space should lead to optic nerve sheath distension and disk edema. Therefore, elevated pressure gradient across the lamina cribrosa caused by chronic, gently elevated ICP should finally cause posterior-globe flattening, disk edema, choroid folds, and a hyperoptic shift (1).

Zhang et al (1) explain ICP regulation by the classic hypothesis of cerebrospinal fluid (CSF) physiology, which describes CSF circulation as a third circulation (1-5). However, we believe that the presented hypothesis of VIIP syndrome could be partially elucidated by our new approach to CSF physiology. Based on our CSF physiology hypothesis, another hypothesis of ICP and IOP regulation was derived, in which the total pressure is expressed by the equation (1):

P_o_ = P_v_+FxR

where P_o_ is ICP or IOP, P_v_ is the superior sagittal sinus pressure or episcleral venous pressure; while F and R are the CSF or aqueous humor system formation rate and the outflow resistance (4-6). This equation shows that both pressures directly depend on the CSF secretion, circulation, and absorption, and that the pressures will be higher if there is greater resistance to the CSF circulation and absorption. It should be emphasized that there are no principle differences regarding IOP and ICP regulation explanation.

We have recently abandoned the classic hypothesis of CSF physiology and formed a new one (7-9). The hypothesis of ICP regulation has been derived from the new CSF hypothesis and from experimental results on animals and a phantom, together with theoretical analyses of equations describing fluid mechanics. Hence, the ICP regulation appears not to be a product of CSF formation, circulation, and absorption relationship, as is generally believed (1,4-6), but depends on the laws of fluid mechanics and on the anatomical characteristics inside the cranial and spinal CSF space (10-13). ICP is a hydrostatic pressure, which directly depends on the gravitational force. In the human brain ventricles in the sitting position ICP is about -10 cm H_2_O, while in the lumbar space it is about +60 cm H_2_O (10-13). According to the laws of fluid mechanics, if the gravitation is zero or almost zero, the pressure gradient should disappear (from -10 to +60), and negative ICP values should become positive, which should permanently increase the ventricular ICP (10-13).

Furthermore, our experiments on cats (14) have shown that significant ICP changes caused by changes in body position did not significantly change IOP. Thus, while during a body position shift from horizontal to vertical, the ICP in ventricles decreased significantly, for 21.5 cm H_2_O (from 17.4 to -4.1 cm H_2_O), the IOP decreased insignificantly, for 4.2 cm H_2_O (from 18.5 to 14.3 cm H_2_O). Our results clearly show that in the vertical body position there is a (physiological) gradient between ICP and IOP, predominantly in the eyes.

This means that regulation mechanisms of ICP and IOP are fundamentally different, rather than the same as was traditionally believed (1,4-6). Therefore, micro-gravity, which directly affects ICP, should not have such an effect on IOP. A different way of pressure regulation is likely what causes an altered pressure gradient. Accordingly, a significantly higher ICP should reduce the (physiological) gradient between ICP and IOP.

To conclude, according to the new hypothesis of ICP regulation, an ICP increase is expected in microgravity, and represents an additional confirmation of the new CSF physiology hypothesis. Additionally, due to different regulatory mechanisms of IOP and ICP, a non-physiological gradient between these pressures should exist. However, the etiology of VIIP syndrome is expected to remain only hypothetical as long as human ICP and IOP are not measured in long-lasting microgravity conditions.
